# Effect of a Defective Clamp Loader Complex of DNA Polymerase III on Growth and SOS Response in *Pseudomonas aeruginosa*

**DOI:** 10.3390/microorganisms10020423

**Published:** 2022-02-12

**Authors:** Maria Concetta Spinnato, Alessandra Lo Sciuto, Jessica Mercolino, Massimiliano Lucidi, Livia Leoni, Giordano Rampioni, Paolo Visca, Francesco Imperi

**Affiliations:** 1 Department of Science, Roma Tre University, 00146 Rome, Italy; mariaconcetta.spinnato@uniroma3.it (M.C.S.); alessandra.losciuto@uniroma3.it (A.L.S.); jes.mercolino@stud.uniroma3.it (J.M.); massimiliano.lucidi@uniroma3.it (M.L.); livia.leoni@uniroma3.it (L.L.); giordano.rampioni@uniroma3.it (G.R.); paolo.visca@uniroma3.it (P.V.); 2 IRCCS Fondazione Santa Lucia, 00179 Rome, Italy

**Keywords:** essential gene, conditional mutagenesis, homologous recombination, replication, replication fork stalling, RuvABC, SOS response, specialized DNA polymerases

## Abstract

DNA polymerase III (Pol III) is the replicative enzyme in bacteria. It consists of three subcomplexes, the catalytic core, the β clamp, and the clamp loader. While this complex has been thoroughly characterized in the model organism *Escherichia coli*, much less is known about its functioning and/or its specific properties in other bacteria. Biochemical studies highlighted specific features in the clamp loader subunit ψ of *Pseudomonas aeruginosa* as compared to its E. *coli* counterpart, and transposon mutagenesis projects identified the ψ-encoding gene *holD* among the strictly essential core genes of *P*. *aeruginosa*. By generating a *P*. *aeruginosa holD* conditional mutant, here we demonstrate that, as previously observed for *E*. *coli holD* mutants, HolD-depleted *P*. *aeruginosa* cells show strongly decreased growth, induction of the SOS response, and emergence of suppressor mutants at high frequency. However, differently from what was observed in E. *coli*, the growth of *P*. *aeruginosa* cells lacking HolD cannot be rescued by the deletion of genes for specialized DNA polymerases. We also observed that the residual growth of HolD-depleted cells is strictly dependent on homologous recombination functions, suggesting that recombination-mediated rescue of stalled replication forks is crucial to support replication by a ψ-deficient Pol III enzyme in *P*. *aeruginosa*.

## 1. Introduction

Genome replication is crucial for the transmission of genetic instructions that drive life processes in all organisms. Bacteria can replicate DNA at very high speed and with remarkable fidelity, through the activity of a complex protein machinery named replisome [1,2,3]. This machinery consists of a helicase (DnaB), a primase (DnaG), single-stranded DNA binding protein (SSB), and the DNA Polymerase III (Pol III) holoenzyme, also known as replicase, which is the main replicative DNA polymerase in bacterial cells. The Pol III enzyme is endowed with important properties that are required for efficient DNA replication, including a rapid elongation rate, high processivity, and tolerance to physiological salt concentrations [4,5].

**Figure 1:**
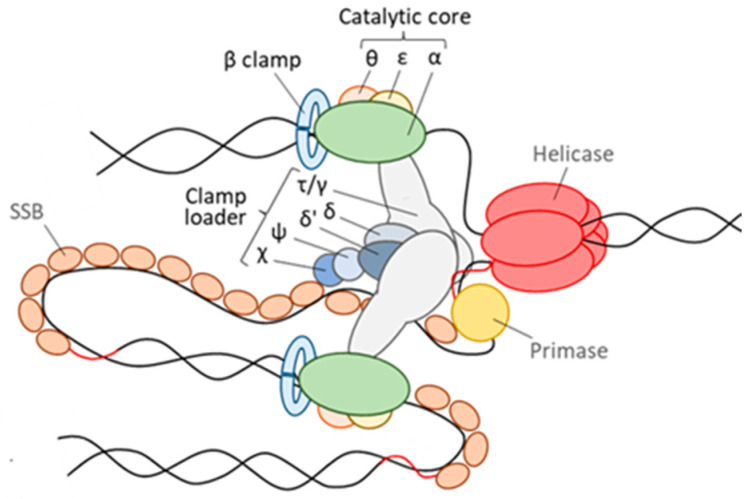
Schematic of the prototypical Pol III holoenzyme. The three subcomplexes of the Pol III holoenzyme, namely the catalytic core, the β clamp and the clamp loader, and their protein subunits are shown in the figure. The bacterial replisome also includes a helicase, which unwinds the double-stranded DNA (dsDNA), a primase, which synthesizes the short RNA primers (in red), and SSB proteins, which bind to and protect single-stranded DNA (ssDNA) filaments.

Pol III can be divided into three subcomplexes, namely the catalytic core, the β clamp, and the clamp loader [4,5] (Figure 1). Extensive genetic and biochemical studies, most of which performed in *Escherichia coli* as model organism or using the *E. coli* replicase in vitro, have elucidated the architecture and function of the three subcomplexes and the importance of their different subunits. The Pol III core is composed of the subunits α (the polymerase catalytic subunit), ε (the proofreading 3′-5′ exonuclease), and θ, a nonessential subunit that appears to be specific to *Enterobacteriaceae* [4,5,6]. The ring-shaped β clamp (or sliding clamp), formed by a dimer of the β subunit, encircles the double-stranded DNA (dsDNA), slides along dsDNA, and tethers the Pol III core to the DNA, hence significantly increasing the processivity of DNA replication [7,8]. The clamp loader (also known as the DnaX complex) is responsible for loading the sliding clamp onto DNA. In *E. coli* it consists of three monomers of the γ and/or τ subunits (both encoded by the *dnaX* gene through a programmed translational frameshift) and one monomer each of the δ, δ′, χ and ψ subunits, although the complex τ/γ_3_δδ′ appears to be the minimal module sufficient for β clamp loading [8]. The Pol III holoenzyme contains two copies of the Pol III core, each responsible for replication of one DNA strand, which are connected by the attachment to a single clamp loader [9] (Figure 1). The accessory clamp loader subunits χ and ψ form a tight 1:1 complex involved in tethering the clamp loader to the SSBs, which coat single-stranded DNA (ssDNA) at the lagging strand to protect it from degradation, thus increasing the efficiency of lagging strand synthesis. Moreover, the χψ complex contributes to stabilizing the clamp loader and increasing its affinity for the β clamp [8].

The protein subunits of the replisome are overall highly conserved among bacterial phyla [10,11]. However, relatively few studies have investigated how they work in bacteria other than *E. coli* or whether they are endowed with different functions with respect to their *E*. *coli* homologs. For instance, work aimed at characterizing the in vitro biochemical properties of the replisome machinery of the human pathogen *Pseudomonas aeruginosa* revealed that this bacterium has a highly divergent ψ subunit that cannot be identified by homology search and that is almost twice as large as the *E. coli* ψ subunit [12,13]. At the functional level, the *P*. *aeruginosa* χψ complex appears to play a more significant role than its *E*. *coli* counterpart, as in vitro it is required for efficient clamp loader activity under physiological salt concentrations and can directly bind to ssDNA via an additional *N*-terminal domain of ψ, both properties being absent from the *E. coli* χψ complex [13,14].

In agreement with the accessory role of ψ in clamp loader activity in vitro, the ψ encoding gene *holD* was deemed as a nonessential gene in *E*. *coli* [15,16,17], even if a recent transposon mutagenesis project argued against this notion [18]. *E. coli holD* deletion mutants have been obtained and are characterized by frequent replication fork arrests, hyperactivation of the SOS response, appearance of suppressor mutants at high frequency, and severe growth defects, that are mainly caused by the overexpression of SOS-induced specialized DNA polymerases [16,17,19].

Several independent transposon mutagenesis studies aimed at determining the “essentialome” (i.e., the complete set of genes that are individually essential in a given organism) of *P*. *aeruginosa* identified *holD* among the genes that were never disrupted by transposon insertions in different *P*. *aeruginosa* strains and under several culture conditions [20,21,22,23,24,25], strongly suggesting that the HolD protein plays a crucial role in *P*. *aeruginosa* physiology. Notably, *P. aeruginosa* insertion mutants in the *holC* gene, which encodes the χ subunit of the clamp loader, were instead obtained in most studies and/or conditions [20,21,22,23,24,25].

To confirm the essentiality of *holD* and to investigate the effect of HolD depletion in *P*. *aeruginosa*, in this study we report the generation of a *holD* conditional mutant and demonstrate that, as previously observed for *E. coli holD* mutants [16,17,19], HolD-depleted *P*. *aeruginosa* cells are characterized by a strongly decreased growth, constitutive induction of the SOS response and frequent emergence of suppressor mutants. However, different from what was observed in *E*. *coli* [17], the growth of *P*. *aeruginosa* cells lacking HolD cannot be restored by the inactivation of specialized DNA polymerases. Moreover, we observed that a functional homologous recombination pathway is essential for residual growth of HolD-depleted *P*. *aeruginosa* cells, suggesting that the reactivation of stalled replication forks through recombination plays a pivotal role during replication by a ψ-deficient Pol III enzyme in *P*. *aeruginosa*.

## 2. Materials and Methods

### 2.1. Bacterial Strains and Growth Media

Bacterial strains used in this study are listed in Table S1. Bacteria were cultured in Lysogeny Broth, Lennox formulation (LB; Acumedia, Neogen, Milan, Italy) for genetic manipulation, while growth assays were performed in Mueller–Hinton broth (MH; Difco, Becton Dickinson, Milan, Italy). When specified, growth media were supplemented with rhamnose or IPTG at the indicated concentrations. When required, antibiotics were added at the following concentration for *E*. *coli* (the concentration used for *P*. *aeruginosa* are shown between brackets): ampicillin 100 µg/mL, nalidixic acid 20 µg/mL, chloramphenicol 30 µg/mL (350 µg/mL), tetracycline 12 µg/mL (50–100 µg/mL).

### 2.2. Generation of Plasmids

Recombinant DNA procedures have been described elsewhere [26]. All DNA fragments for cloning were amplified by PCR using the Q5 Hot Start High-Fidelity DNA Polymerase (New England Biolabs, Euroclone, Milan, Italy) and the genomic DNA of *P*. *aeruginosa* PAO1 as the template. Primers and restriction enzymes used for cloning are described in Table S2. All the constructs generated in this study were verified by restriction analysis and DNA sequencing and are described in Table S1.

The integration-proficient construct pJM253*holD* was generated by directionally cloning the coding sequence of the *holD* gene into the mini-CTX1 derivative pJM253 [27] downstream of the *rhaRS*-P_rhaBAD_ regulatory element.

The deletion mutagenesis constructs pDM4Δ*holD*, pDM4Δ*polA*, pDM4Δ*polB*, pDM4Δ*dinB* and pDM4Δ*imuC* were obtained by directionally cloning two DNA fragments of about 500 bp each, corresponding to the upstream and downstream regions of the coding sequence of each gene of interest, into pBluescript II KS+ (Table S1), followed by DNA sequencing and subcloning of the entire insert encompassing the upstream and downstream regions of the gene of interest into the suicide vector pDM4 [28]. To obtain the construct for the deletion mutagenesis of *ruvCAB* genes (pDM4Δ*ruvCAB*), two DNA fragments corresponding to the region upstream of *ruvC* and the region downstream of *ruvB* were directionally cloned into pBluescript II KS, checked by sequencing and then subcloned into pDM4. The construct pDM4Δ*recA* has been described elsewhere [29].

To generate the construct for IPTG-inducible expression of FtsZ fused to GFP (pME*ftsZ-GFP*), the *ftsZ* coding sequence without the stop codon was amplified and cloned into pBluescript II KS. The GFP coding sequence was amplified from pPS858 [30] and cloned in frame downstream of the *ftsZ* coding sequence. Then, the resulting *ftsZ-GFP* fusion gene was verified by sequencing and subcloned into the shuttle vector pME6032 [31] downstream of the P_tac_ promoter.

The expressing constructs pME*polA*, pME*polB*, pME*dinB* and pME*imuBC* (Table S1) were generated by cloning the PCR-amplified gene(s) of interest, without the endogenous promoter, into the IPTG-inducible shuttle vector pME6032 downstream of the P_tac_ promoter. The pME6032 derivatives were introduced into *P*. *aeruginosa* by transformation using chemically competent cells.

### 2.3. Generation of Mutant Strains

Deletion mutants were obtained by homologous recombination using the *sacB*-based suicide pDM4 derivatives, as previously described [32].

To obtain the *P*. *aeruginosa* PAO1 *holD* conditional mutant, the *holD* coding sequence under the control of the rhamnose-dependent regulatory element in pJM253*holD* was integrated into the *attB* neutral site of the *P*. *aeruginosa* PAO1 chromosome, and the mini-CTX1 plasmid backbone was excised by Flp-mediated site-specific recombination as described [33]. Then, in-frame deletion of the endogenous copy of *holD* was obtained under permissive condition (i.e., growth in the presence of 0.01% rhamnose) using the *sacB*-based suicide construct pDM4Δ*holD*, as previously described [32].

### 2.4. Growth Assays

Growth assays in liquid cultures were performed in 96-well microtiter plates (200 µL of medium in each well) or in flasks at 37 °C and vigorous shaking (200 rpm). Growth was measured as the optical density at 600 nm wavelength (OD_600_) of bacterial cultures in a Tecan Spark 10M microtiter plate reader for microtiter plates or of appropriate dilutions in sterile growth medium in a spectrophotometer for flask cultures. To obtain HolD-depleted cells of the *holD* conditional mutant derivatives that did not grow in the absence of the inducer, a previously described dual-refresh culturing strategy was used [34]. Briefly, cells were cultured overnight in the presence of 0.01% rhamnose and then refreshed at high cell density (1:20 dilution) in the absence of rhamnose, or in the presence of 0.01% rhamnose as control, cultured for 2 h and then refreshed again (1:30 dilution) in the same medium. HolD-depleted cells were collected as soon as a growth defect was observed with respect to control cultures.

Growth assays on solid media were performed by directly streaking bacterial suspensions normalized in saline at an OD_600_ = 1, obtained from late-exponential cultures grown in the presence of 0.01% rhamnose, onto MH solidified with 1.5% agar with a 1-μL inoculation loop. Plating efficiency was investigated by spotting 5 µL of serial ten-fold dilutions from the same bacterial suspensions onto MH agar plates supplemented or not with rhamnose. The frequency of fast-growing colonies for the *holD* conditional mutant and its derivatives was determined by plating 50 µL of the bacterial suspensions normalized at OD_600_ = 1 onto MH agar plates without rhamnose and by dividing the number of colonies obtained on these plates to the number of colony forming units (CFU) determined by plating ten-fold serial dilutions onto MH agar plates supplemented with 0.01% rhamnose.

### 2.5. Gene Expression Analysis

The expression level of selected SOS genes and of DNA polymerase genes was determined by quantitative reverse transcription PCR (qRT-PCR). Bacterial cells were harvested by centrifugation and treated with RNAprotect Bacteria Reagent (Qiagen, Milan, Italy). RNA was purified using the RNeasy Mini kit (Qiagen), treated with DNase, and re-purified with the RNeasy MinElute Cleanup kit (Qiagen). cDNA was reverse transcribed from 100 ng of RNA with Prime Script RT Reagent Kit (Takara, Unimed Scientifica, Rome, Italy). Appropriate dilutions of the cDNA were used as template for qRT-PCR in a AriaMx Real-Time PCR System (Agilent Technologies, Rome, Italy) using TB Green Premier EX Taq master mix (Takara) and the primers listed in Table S2. Relative gene expression with respect to the housekeeping gene *rpoD* was calculated using the 2^−ΔΔCt^ method [35].

### 2.6. Confocal Microscopy

Cells carrying the constructs pME*ftsZ-GFP* were cultured in MH supplemented with 0.003 mM IPTG in the absence or presence of 0.01% rhamnose, as described above. Cells were collected by centrifugation, washed and suspended in saline in the presence of the fluorescent dye FM4-64 (10 μg/mL) [36], and incubated at 37 °C for 1 h. Then, the excess of FM4-64 was removed by an additional wash in saline. Five µL of the bacterial cell suspensions were spotted on a microscope glass slide overlaid with 0.5% agarose and imaged with a NikonA1+ confocal laser scanning microscope equipped with an Apo TIRF 100× oil immersion objective (NA 1.49). The 488 and 561 nm laser lines were employed for the GFP and FM4-64 excitation, respectively. The emission bandwidths at 500–540 nm and 600–720 nm were used for the GFP and FM4-64 detection, respectively. The images were acquired at a sampling dimension of 512 × 512 pixels and were deconvoluted using the NIS-Elements software (Nikon, Amsterdam, The Netherlands), using default parameters.

### 2.7. Statistical Analysis

Statistical analysis was performed with the software GraphPad Instat, using one-way analysis of variance (ANOVA) followed by Tukey-Kramer multiple comparison test.

## 3. Results

### 3.1. HolD Depletion Strongly Delays P. aeruginosa Growth

To verify the essentiality of the *P*. *aeruginosa holD* orthologue, we first tried to generate a *holD* clean deletion mutant in the reference strain PAO1 (annotated as PA4679). However, despite the numerous attempts, we were unable to delete the *holD* gene from the *P*. *aeruginosa* PAO1 chromosome. We therefore generated an *holD* conditional mutant through a previously described strategy [27], based on the introduction of a copy of the *holD* coding sequence under a rhamnose dependent promoter in a neutral site (*attB*) of the chromosome, followed by in-frame deletion of the endogenous *holD* gene under permissive conditions (presence of rhamnose in the growth medium). Notably, while the coding sequence of *holD* in PAO1 and other *P*. *aeruginosa* strains is annotated as a 702-bp sequence, encoding a 233-aa polypeptide (www.pseudomonas.com; accessed on 2 January 2021), Jarvis and co-workers have demonstrated that the ψ subunit of the *P*. *aeruginosa* Pol III that is functional in vitro is 278-aa long, deriving from a translational start site located 135 nt upstream of the annotated one for PA4679 [13] (Figure S1). In line with the finding of Jarvis et al., we failed to generate the conditional mutant by using a rhamnose-dependent PA4679 coding sequence corresponding to the 233-aa polypeptide (data not shown), while we readily obtained the *holD* conditional mutant when the longer coding sequence was introduced under the rhamnose-dependent promoter into the *P*. *aeruginosa* PAO1 chromosome. This evidence indirectly confirms that the 278-aa long protein is the functional *P*. *aeruginosa* HolD also in vivo.

**Figure 2:**
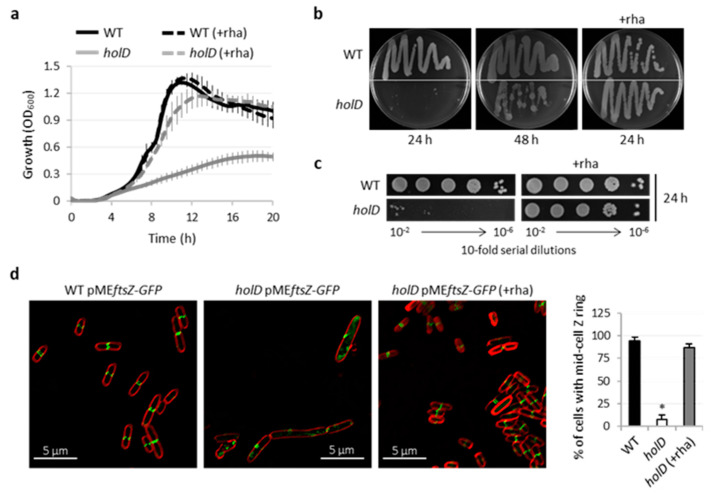
Effect of HolD depletion in *P*. *aeruginosa*. (**a**) Growth curves of the *P*. *aeruginosa* wild-type (WT) strain PAO1 and the *holD* conditional mutant (*holD*) at 37 °C in MH supplemented or not with 0.01% rhamnose (+rha). Data are the mean (±SD) of three independent assays. (**b**) Growth and (**c**) plating efficiency of PAO1 and *holD* on MH agar plates supplemented or not with 0.01% rhamnose (+rha) after 24 and/or 48 h of incubation at 37 °C. (**d**) Confocal microscopy images of PAO1 and *holD* expressing the fusion protein FtsZ-GFP cultured in the presence of 0.003 mM IPTG and, when indicated, 0.01% rhamnose (+rha), and stained with the membrane dye FM4-64. The graph on the right shows the percentage of cells showing mid-cell localization of the FtsZ-GFP fusion protein for PAO1, *holD* and *holD* cultured in the presence of rhamnose (+rha). Data are the mean (±SD) of three independent experiments, and at least 50 cells were analyzed per sample and experiment. The asterisk indicates a statistically significant difference (*p* < 0.05) with respect to the WT strain. Images in panels (**b**–**d**) are representative of at least three independent experiments.

The growth of the *holD* conditional mutant was confirmed to be strongly dependent on rhamnose, and a marked reduction of growth kinetics and yields with respect to the wild-type was observed when the mutant was cultured in the absence of rhamnose (Figure 2a), explaining the failure in generating *holD* knock-out mutants in *P*. *aeruginosa* during transposon mutagenesis and Tn-seq studies [20,21,22,23,24,25]. Notably, HolD-depleted cells showed some residual growth under non-inducing conditions, and this was confirmed by streaking the mutant on agar plates, where relevant growth was observed after prolonged incubation (>30 h) in the absence of rhamnose (Figure 2b). Growth assays on plates also suggested the presence of fast-growing colonies in the absence of rhamnose, here defined as colonies that were clearly visible within the first 24 h of growth, when the bulk of the population was unable to develop detectable colonies (Figure 2b). Spotting of serial dilutions of bacterial cultures grown in the presence of rhamnose onto agar plates supplemented or not with the inducer confirmed the emergence of such fast-growing cells under non-inducing conditions, with an apparent frequency of about 10^−4^ in the bacterial population (Figure 2c). The lack or depletion of Pol III subunits hampers or delays DNA replication, resulting in elongated or filamentous bacterial cells [37,38,39]. Moreover, since cell division is temporally and spatially dependent on chromosome replication, a defective replisome activity can result in inhibition or mislocalization of the divisome machinery [37,40,41]. To verify if these phenotypes were also associated with HolD depletion in *P*. *aeruginosa*, we generated and introduced into the wild-type and the *holD* conditional mutant a plasmid (pME*ftsZ-GFP*) for the IPTG-inducible expression of a fusion protein between the green fluorescent protein (GFP) and FtsZ, a pivotal divisome protein that, by polymerizing into the Z ring, defines the division site and recruits the downstream divisome proteins that drive cell constriction [42,43]. Preliminary experiments were performed to identify the optimal IPTG concentration that allows detectable expression of the FtsZ-GFP fusion protein without affecting bacterial growth and cell morphology (Figure S2). Wild type cells and *holD* conditional mutant cells were then cultured with the selected IPTG concentration (0.003 mM) and subjected to confocal microscopy analysis. As shown in Figure 2d, wild-type cells, as well as conditional mutant cells cultured in the presence of rhamnose, showed a normal rod shape with the Z ring correctly localized at the mid cell, in line with the septum position in dividing cells. In contrast, conditional mutant cells grown under non-inducing conditions appeared elongated, without a definite mid-cell Z ring and with a more diffused FtsZ-GFP fluorescence inside the cell (Figure 2d), suggesting that HolD depletion leads to a delayed and/or defective chromosome replication that, in turn, hampers the proper assembly of the divisome machinery.

### 3.2. The SOS Response Is Induced in HolD-Depleted P. aeruginosa Cells

**Figure 3:**
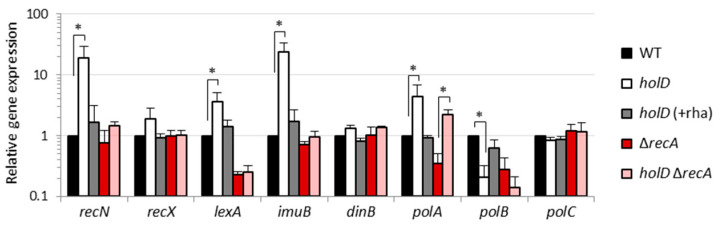
HolD depletion induces the SOS response and affects the expression of DNA polymerases. Expression levels of the SOS genes *recN*, *recX*, *lexA*, and *imuB* and of the DNA polymerase genes *dinB*, *polA*, *polB*, and *polC*, determined by qRT-PCR, in the *P*. *aeruginosa* wild-type (WT) strain PAO1, the *holD* conditional mutant (*holD*), the Δ*recA* mutant, and the *holD* Δ*recA* conditional mutant, cultured in the absence or in the presence of 0.01% rhamnose (+rha). Relative gene expression is shown as fold induction with respect to the W.T. Data represent the mean (±SD) of three independent experiments performed in triplicate. The asterisks indicate a statistically significant difference (*p* < 0.05) in *holD* and *holD* Δ*recA* with respect to the cognate HolD-replete strains WT and Δ*recA*, respectively.

Poor functioning or reduced activity of the Pol III can impede the replication fork progression, and this often results in an increased frequency of fork stalling and accumulation of ssDNA gaps [16,44]. The ssDNA is known to activate the RecA protein and induce the SOS response, a global stress response to DNA damages that induces low-fidelity DNA repair mechanisms and error-prone specialized DNA polymerases, generally leading to increased mutagenesis rates [45,46]. Accordingly, the SOS response was found to be constitutively induced in *E*. *coli* cells with a defective clamp loader complex of Pol III due to the lack of the ψ subunit [17]. Given that the emergence of fast-growing variants among HolD-depleted *P*. *aeruginosa* cells is suggestive of suppressor mutants, which seems to occur with a relatively high frequency (Figure 2c), we wondered whether HolD depletion leads to the induction of the SOS response in *P*. *aeruginosa* as well. Therefore, the expression levels of well-known SOS genes (*recN*, *recX*, *lexA* and *imuB*) [47,48,49] were compared by qRT-PCR between the wild-type and the *holD* conditional mutant cultured in the presence or in the absence of rhamnose. We observed an increase in the mRNA levels of the four SOS genes in HolD-depleted cells as compared to wild-type cells, although the increase was not statistically significant for *recX* (Figure 3). As expected, the expression of these genes was overall restored to wild-type levels when the *holD* conditional mutant was cultured in the presence of rhamnose (Figure 3). This result confirms that a clamp loader complex deficient of HolD also induces the SOS response in *P*. *aeruginosa*.

### 3.3. RecA Sustains the Residual Growth of HolD-Depleted, P. aeruginosa Cells

**Figure 4:**
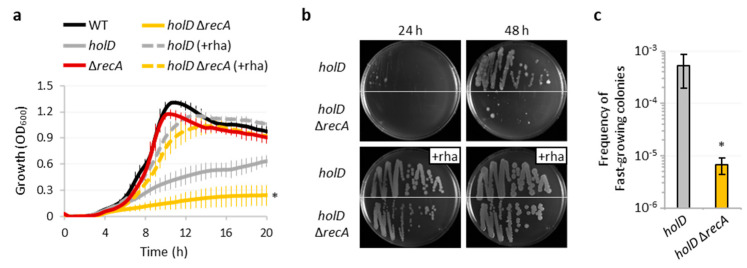
RecA is crucial for the residual growth of HolD-depleted *P*. *aeruginosa* cells. (**a**) Growth curves of the *P*. *aeruginosa* wild-type (WT) strain PAO1, the *holD* conditional mutant (*holD*), the Δ*recA* mutant, and the *holD* Δ*recA* conditional mutant at 37 °C in MH supplemented or not with 0.01% rhamnose (+rha). (**b**) Growth of PAO1, *holD*, Δ*recA*, and *holD* Δ*recA* strains on MH agar plates supplemented or not with rhamnose after 24 and 48 h of incubation at 37 °C. (**c**) Frequency of fast-growing colonies obtained on MH agar plates without rhamnose for the *holD* and *holD* Δ*recA* conditional mutants. Data are the mean (±SD) of at least three independent assays. The asterisks indicate a statistically significant difference (*p* < 0.05) between the *holD* and *holD* Δ*recA* conditional mutants. The images in panel (**b**) are representative of several experiments giving similar results.

The observed induction of SOS genes in *P*. *aeruginosa* cells depleted of HolD suggests that the SOS response could play a role in the residual growth of the *holD* conditional mutant and/or the emergence of fast-growing variants under non-inducing conditions (Figure 2), by triggering the expression of specialized DNA polymerases that can either partially sustain replication in Pol III defective cells or increase the mutation rates. To explore this hypothesis, we deleted the *recA* gene in the *holD* conditional mutant and in the wild-type as control, thus obtaining strains unable to induce the SOS response. The lack of RecA did not significantly affect the growth of the wild-type strain, while it almost abrogated the residual growth of HolD-depleted cells, both in liquid cultures and on agar plates (Figure 4a,b). The qRT-PCR analysis showed that the induction of SOS genes observed in HolD-depleted cells was nearly completely abolished upon deletion of *recA* (Figure 3), confirming that the SOS response is inactive in cells lacking RecA. It is interesting to note that, except for *lexA*, which is one of the most responsive SOS genes in *P*. *aeruginosa* [47,49], the expression of other SOS genes was not affected by *recA* inactivation in the wild-type background (Figure 3), indicating that the SOS response is poorly induced in the wild-type strain under the growth conditions used in our study. Since fast-growing colonies still emerged when the *holD* Δ*recA* conditional mutant was streaked onto agar plates in the absence of the inducer (Figure 4b), we compared the frequency of these variants between SOS response-proficient or -deficient HolD-depleted cells. The *holD* Δ*recA* conditional mutant showed an 80-fold reduction in the frequency of fast-growing colonies with respect to its RecA-proficient parental strain (Figure 4c). This implies that RecA is required for most of the genetic and/or adaptive mechanisms involved in the emergence of these variants, even if they can arise with a relevant frequency (>10^−6^) also in a RecA-independent manner.

### 3.4. Specialized DNA Polymerases Marginally Affect the Viability of HolD-Depleted P. aeruginosa Cells

The induction of the SOS response and the importance of RecA for growth and emergence of fast-growing variants upon HolD depletion support the hypothesis that specialized DNA polymerases could be involved in the viability of HolD-depleted *P. aeruginosa* cells. The three specialized DNA polymerases of *E. coli*, Pol II (PolB), and the error-prone enzymes Pol IV (DinB) and Pol V (UmuBC), are all induced by the SOS response [50]. *P. aeruginosa* also has three specialized DNA polymerases, namely PolB, DinB and ImuBC, a functional analog of Pol V [51], although *umuBC* orthologues have been recently identified in few clinical isolates [52]. While the operon that encodes ImuBC definitely belongs to the SOS regulon [47,53,54], as confirmed in this work (Figure 3), the role of the SOS response in the control of *polB* and *dinB* gene expression in *P. aeruginosa* is less clear, with inconsistent results obtained in different studies [47,55]. Thus, we first analyzed by qRT-PCR whether these genes were induced in HolD-depleted cells in the presence and in the absence of a functional SOS response. The genes *polA* and *polC*, encoding the catalytic subunits of the replicative enzymes Pol I and Pol III, respectively, were included in the analysis as controls. No increase in the mRNA levels of *dinB* and *polC* was observed in HolD-depleted *P*. *aeruginosa* cells, irrespective of the presence or the absence of RecA (Figure 3). In contrast, a significant downregulation of *polB* and upregulation of *polA* were observed in HolD-depleted cells, in a RecA-independent manner (Figure 3).

**Figure 5:**
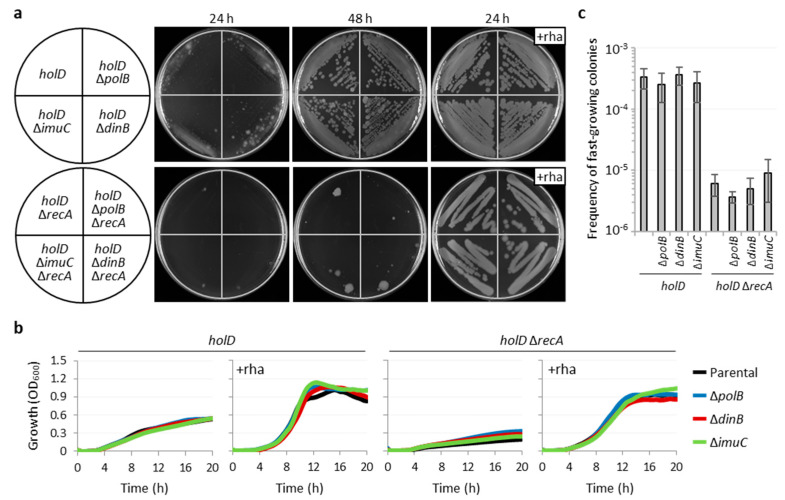
Specialized DNA polymerases do not account for residual growth and emergence of fast-growing variants in the *holD* conditional mutant. (**a**) Growth of the *P*. *aeruginosa holD* conditional mutant (*holD*), the *holD* Δ*recA* conditional mutant and their cognate *polB*, *dinB* or *imuC* deletion mutants on MH agar plates after 24 and 48 h of incubation at 37 °C. Images are representative of three independent assays. (**b**) Growth curves of the same strains described in panel (**a**) in MH at 37 °C. Data are the mean of three independent assays. When indicated, liquid and solid media were supplemented with 0.01% rhamnose (+rha). (**c**) Frequency of fast-growing colonies obtained on MH agar plates without rhamnose for the same strains described in panels (**a**,**b**). Data are the mean (±SD) of five independent assays.

To verify whether any of the specialized polymerases contributes to the growth of cells lacking HolD, we generated *holD* and *holD* Δ*recA* conditional mutants also deleted in *polB*, *dinB* or *imuC*. The lack of *polB*, *dinB* or *imuC* did not impair the growth of the *holD* conditional mutant and did not rescue the growth of the *holD* Δ*recA* conditional mutant under non-inducing conditions (Figure 5a,b), implying that the residual growth of HolD-depleted cells is not dependent on the presence of any specialized polymerase. As expected, the lack of specialized polymerases did not inhibit the growth of HolD-proficient wild-type and Δ*recA* cells, either (Figure S3). Additionally, the frequency of fast-growing variants obtained for the *holD* conditional mutants deficient in specialized polymerases was comparable to that of the corresponding parental strains (Figure 5c), indicating that the activity of error-prone polymerases is not the main mechanism that accounts for the emergence of these fast-growing variants.

Several attempts to delete the *polA* gene were unsuccessful in all of our genetic backgrounds, suggesting that PolA deficiency strongly affects *P*. *aeruginosa* growth and/or cell viability. While this would be reasonable considering the involvement of PolA in removing RNA primers and filling the resulting gaps during replication [56], our inability to generate Δ*polA* mutants in *P*. *aeruginosa* is in contrast with the observation that viable knock-out mutants in *polA* have been obtained in random transposon mutagenesis studies [20,21,22,23], implying that this gene could be dispensable in *P*. *aeruginosa*. While the relevance of PolA in *P*. *aeruginosa* deserves further investigation, we can reasonably rule out that this DNA polymerase is responsible for the RecA-dependent residual growth of HolD-depleted cells, as it was found to be induced upon HolD depletion in both RecA-proficient and -deficient cells (Figure 3), although only the former showed relevant growth in the absence of rhamnose (Figure 4).

To further investigate the correlation between PolA activity and residual growth of HolD-depleted cells, the PolA enzyme was overexpressed from an IPTG-inducible plasmid (pME*polA*), and the growth of HolD-depleted cells was then monitored. As a control, the specialized DNA polymerases PolB, DinB and ImuBC were also individually overexpressed from the same plasmid vector. Except for pME*imuBC*, the overexpression of all DNA polymerases inhibited the growth of HolD-depleted cells with respect to the empty plasmid controls, and none of them rescued the growth of HolD-depleted RecA-deficient cells (Figure S4). The inhibitory effect of polymerase-expressing constructs was also observed in HolD-depleted cells cultured without IPTG (Figure S4), plausibly because of leaky gene expression from the pME6032 plasmid in the absence of the inducer [57]. This effect appears to be dependent on HolD depletion, as the overexpression of the DNA polymerases marginally affected the growth of wild-type and Δ*recA* cells (Figure S4). Besides highlighting that HolD-deficient *P*. *aeruginosa* cells are highly sensitive to an unbalanced expression of specialized DNA polymerases, this experiment and, in particular, the results obtained with the pME*polA* construct corroborate that the observed induction of *polA* upon HolD depletion (Figure 3) likely does not account for the residual growth of HolD-depleted *P*. *aeruginosa* cells.

### 3.5. Homologous Recombination Is Essential for HolD-Depleted P. aeruginosa Cells

In addition to its role in triggering the SOS response, RecA is also crucial for homologous recombination. Indeed, RecA forms nucleoprotein filaments with ssDNA and is responsible for the invasion and scanning of dsDNA to recognize homologous sequences [58]. While homologous recombination has originally been investigated for its importance during horizontal gene transfer, it is now clear that the main function of this process is to support chromosome replication by repairing double-strand breaks and restarting stalled replication forks originating from DNA lesions and/or defects in the replisome activity [59,60].

**Figure 6:**
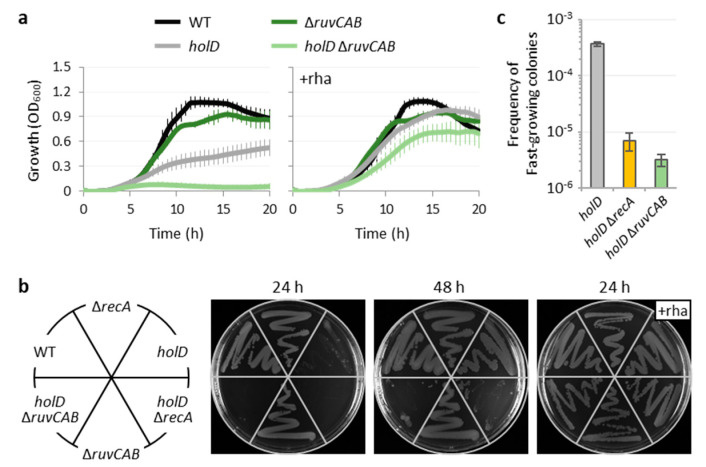
Homologous recombination is responsible for the residual growth of HolD-depleted *P. aeruginosa* cells. (**a**) Growth curves of the *P*. *aeruginosa* wild-type (WT) strain PAO1, the *holD* conditional mutant (*holD*), and the cognate *recA* and *ruvCAB* deletion mutants in MH at 37 °C. Data are the mean of three independent assays. (**b**) Growth on MH agar plates of the same strains described in panel (**a**) after 24 and 48 h of incubation at 37 °C. Images are representative of several independent experiments. When indicated, liquid and solid media were supplemented with 0.01% rhamnose (+rha). (**c**) Frequency of fast-growing colonies obtained on MH agar plates without rhamnose for the same strains described in panels (**a**,**b**). Data are the mean (±SD) of five independent assays.

To verify whether homologous recombination, rather than the activation of the SOS response, is required to sustain the growth of *P*. *aeruginosa* with a poorly functional HolD-deficient Pol III enzyme, we deleted the entire *ruvCAB* operon in the *holD* conditional mutant and in the wild-type strain. This operon encodes for three enzymes (RuvABC, also known as the resolvasome) that catalyze the resolution of Holliday junctions, i.e., the last step of homologous recombination, without being directly involved in the activation of the SOS response [61,62]. The *holD* Δ*ruvCAB* conditional mutant showed a growth phenotype reminiscent of the *holD* Δ*recA* conditional mutant, although the defects were somehow more exacerbated. Indeed, the growth was basically abrogated in the absence of rhamnose, and the frequency of fast-growing cell variants was further reduced with respect to the *holD* Δ*recA* mutant (Figure 6). It is interesting to note that *ruvCAB* deletion in the wild-type background only caused a moderate defect in growth at the onset of the stationary phase (Figure 6a), in line with what was previously observed for the Δ*recA* mutant (Figure 4a). This suggests that neither replication fork reversal nor recombinational DNA repair are required for normal replication under the conditions used in our study or that alternative pathway(s) may compensate for the lack of RuvABC in HolD-proficient *P*. *aeruginosa* cells. In contrast, the growth of the *holD* conditional mutant deficient in Ruv proteins was also significantly impaired under *holD*-inducing conditions (Figure 6a). A less evident growth defect in the presence of rhamnose was observed for the *holD* Δ*recA* and, to a lesser extent, the *holD* conditional mutant (Figure 2a and Figure 4a). Overall, these data suggest that replication might slightly be hampered when *holD* is induced in a rhamnose-dependent non-physiological manner, and that this could result in a higher frequency of replication fork stalling. This would also explain why replication and growth are more closely dependent on homologous recombination proteins in *holD* conditional mutants, as compared to the wild-type strain.

## 4. Discussion

Most of the information available about DNA replication in bacteria and the functional properties of Pol III, the main replicative polymerase involved in this process, derives from studies performed in *E*. *coli*. This work originated from the observation that the clamp loader subunit ψ of Pol III, which was proposed to be dispensable for *E*. *coli* growth and, thus, for Pol III activity in *E*. *coli* cells [15,16,17], appears to belong to the class of strictly essential genes in *P*. *aeruginosa* [20,21,22,23,24,25].

The in vitro biochemical characterization of the *P*. *aeruginosa* ψ subunit (HolD) highlighted important differences with respect to its *E*. *coli* counterpart, such as larger size, ability to directly bind ssDNA, and essentiality for efficient clamp loader activity under physiological salt concentrations [13,14]. Nevertheless, here we demonstrate that the phenotypes of *P*. *aeruginosa* cells depleted of HolD mirror those of *E. coli holD* mutants. Indeed, as previously observed in *E*. *coli* [16,17,19], HolD-depleted *P*. *aeruginosa* cells showed strongly reduced growth, constitutive activation of the SOS response, and frequent emergence of putative suppressor mutants (Figure 2, Figure 3 and Figure 4). In addition, we found that HolD-depleted *P*. *aeruginosa* cells appear elongated with mislocalization of the Z ring (Figure 2), consistent with the intimate link between chromosome replication and cell division [37,40,41]. These features are suggestive of a defective replisome activity, which is generally associated with an increased frequency of stalled replication forks [16,44]. Accordingly, we observed that deletion of genes required for homologous recombination (*recA* or *ruvCAB*) abolishes the residual growth of HolD-depleted *P*. *aeruginosa* cells (Figure 4 and Figure 6), indicating that recombination-mediated reactivation of arrested replication forks [63] plays a crucial role in fork restarting in *P*. *aeruginosa.*

RecA is a dual-function protein that, besides its role in homologous recombination, is also responsible for the activation of the SOS response, a regulatory circuit activated by DNA damages or replication perturbations that induces the expression of DNA repair systems and specialized error-prone DNA polymerases (also known as translesion DNA polymerases), that can bypass DNA lesions and promote replication of damaged DNA [45,46,64]. In *E*. *coli*, the SOS response contributes to the phenotypes of *holD* mutants, as it has been shown that inactivation of either the SOS-induced DNA polymerases PolB (Pol II) or DinB largely restores the growth of HolD-deficient cells [17]. This suggests that the interference of specialized DNA polymerases with a poorly functional ψ-deficient replicase is the main cause of growth defects in *E*. *coli holD* mutants, in line with the notion that the different DNA polymerases can compete for the β clamp [65]. In contrast, we did not find a similar contribution of specialized DNA polymerases to the essentiality of HolD in *P*. *aeruginosa*. In fact, deletion of each specialized DNA polymerase of *P*. *aeruginosa* (PolB, DinB or ImuBC) did not rescue the growth of HolD-depleted cells, both in the presence or in the absence of a functional homologous recombination pathway (Figure 5). However, we observed a marked sensitivity of *P*. *aeruginosa* cells to the overexpression of PolB and DinB, but not ImuBC, in the absence of HolD (Figure S4), suggesting that also the replicase of *P*. *aeruginosa* is extremely sensitive to these two DNA polymerases when the clamp loader lacks the ψ subunit. Notably, our qRT-PCR analysis showed that *polB* and *dinB* are not induced by the SOS response in *P*. *aeruginosa* (Figure 3), in line with previous transcriptomics data [47]. So, the different effect of *polB* or *dinB* gene inactivation on the growth of HolD-depleted cells between *P*. *aeruginosa* and *E*. *coli* is likely due to the different regulatory circuits that trigger the expression of these polymerases in the two bacteria.

Finally, we observed a high frequency of putative suppressor mutants in *P*. *aeruginosa* when HolD is not expressed (Figure 2, Figure 4, Figure 5 and Figure 6). This was also reported for *E*. *coli* cells with defective clamp loader complex [17,19,66]. Interestingly, the emergence of these suppressor mutants in HolD-depleted *P. aeruginosa* cells does not depend on the error-prone DNA polymerases DinB and ImuBC (Figure 5) but is significantly reduced when the homologous recombination pathway is inactivated (Figure 4 and Figure 6). Previous works have shown that the genetic diversity observed in *P*. *aeruginosa* cells growing as biofilm largely derives from the mutagenic repair of dsDNA breaks (DSBs), and that it is abrogated in transposon insertion mutants deficient in RecA or Ruv proteins [67,68]. By analogy, it could be reasoned that this process promotes the emergence of putative suppressor mutants observed in HolD-depleted *P. aeruginosa* cultures. However, these suppressors were still detected at a relatively high frequency (>10^−6^) even upon deletion of recombination genes *recA* or *ruvCAB*. In *E*. *coli*, where some of the suppressor mutations allowing growth of replicase-defective mutants have been characterized, these mutations can affect a variety of physiological functions besides those directly related to replication, such as ion transport, transcription, and cell metabolism [19,66,69]. If this is true for other bacteria as well, such a high number of possible targets could justify the high frequency of suppressor mutants observed in our *P*. *aeruginosa holD* conditional mutant. Further work is required to verify this hypothesis and to explore the genetic determinants that can rescue the growth of *P*. *aeruginosa* cells with a defective clamp loader complex. Considering the interest in bacterial replication as a potential target for drug discovery and rational design [11], this information would be useful to evaluate the suitability of clamp loader inhibitors and/or to propose alternative strategies to interfere with DNA replication in bacterial pathogens. Reference [70] is cited in the supplementary materials.

## Data Availability

Not applicable.

## Supplementary Materials

The following supporting information can be downloaded at https://www.mdpi.com/article/10.3390/microorganisms10020423/s1, Table S1, Bacterial strains and plasmids used in this study; Table S2, Primers used in this study; Figure S1, *holD* genetic locus and DNA sequence in *P. aeruginosa* PAO1; Figure S2, Growth and confocal microscopy analysis of *P. aeruginosa* PAO1 expressing the fusion protein FtsZ-GFP; Figure S3, Growth of *P. aeruginosa* PAO1, the Δ*recA* mutant and their cognate *polB*, *dinB* or *imuC* deletion mutants; Figure S4, Effect of PolA, PolB, DinB or ImuBC overexpression on the growth of HolD-deficient and -proficient *P. aeruginosa* cells.
