# Supplementary material for: Effect of a Defective Clamp Loader Complex of DNA Polymerase III on Growth and SOS Response in Pseudomonas aeruginosa
Source: Microorganisms. 2022 Feb 12;10(2):423. doi: 10.3390/microorganisms10020423 (PMC8879598; doi:10.3390/microorganisms10020423)
Supplement: Supplementary file 1 [file microorganisms-10-00423-s001.zip › microorganisms-1588675-supplementary.pdf]

Effect of a defective clamp loader complex of DNA polymerase III on growth and SOS response in *Pseudomonas aeruginosa*

Maria Concetta Spinnato, Alessandra Lo Sciuto, Jessica Mercolino, Massimiliano Lucidi, Livia Leoni, Giordano Rampioni, Paolo Visca, and Francesco Imperi

SUPPLEMENTARY MATERIAL

Table S1. Bacterial strains and plasmids used in this study.

Strain or plasmid	Genotype and/or relevant characteristics	Reference or source
P. aeruginosa		
PAO1 (ATCC15692)	Prototroph	American type culture collection
PAO1 *rhaRS*-P_{rhaBAD}*holD*	PAO1 with a rhamnose-inducible additional copy of the *holD* (PA4679) coding sequence inserted into the *attB* neutral site	This work
PAO1 Δ*holD rhaRS*-P_{rhaBAD}*holD*	PAO1 *rhaRS*-P_{rhaBAD}*holD* with an in-frame deletion of the endogenous copy of *holD* (hereafter renamed *holD*)	This work
PAO1 Δ*recA*	PAO1 with an in-frame deletion of *recA* (PA3617)	[29]
PAO1 Δ*polB*	PAO1 with an in-frame deletion of *polB* (PA1886)	This work
PAO1 Δ*dinB*	PAO1 with an in-frame deletion of *dinB* (PA0923)	This work
PAO1 Δ*imuC*	PAO1 with an in-frame deletion of *imuC* (PA0669)	This work
PAO1 Δ*ruvCAB*	PAO1 with a deletion of the *ruvCAB* genes (PA0965-7)	This work
PAO1 Δ*polB* Δ*recA*	PAO1 Δ*polB* deleted of *recA*	This work
PAO1 Δ*dinB* Δ*recA*	PAO1 Δ*dinB* deleted of *recA*	This work
PAO1 Δ*imuC* Δ*recA*	PAO1 Δ*imuC* deleted of *recA*	This work
holD Δ*recA*	*holD* conditional mutant deleted of *recA*	This work
holD Δ*polB*	*holD* conditional mutant deleted of *polB*	This work
holD Δ*dinB*	*holD* conditional mutant deleted of *dinB*	This work
holD Δ*imuC*	*holD* conditional mutant deleted of *imuC*	This work
holD Δ*polB* Δ*recA*	*holD* Δ*polB* deleted of *recA*	This work
holD Δ*dinB* Δ*recA*	*holD* Δ*dinB* deleted of *recA*	This work
holD Δ*imuC* Δ*recA*	*holD* Δ*imuC* deleted of *recA*	This work
holD Δ*ruvCAB*	*holD* conditional mutant deleted of *ruvCAB*	This work
E. coli		
S17.1λ*pir*	*thi pro hsdRhsdM*$^{+}$*recA RP4-2-Tc::Mu-Km::Tn7 λpir,* Sm^R	[70]
**Plasmid**		
pBluescript II KS+	Cloning vector; ColE1 replicon; Ap^R	Stratagene
pDM4	Suicide vector; *sacB, oriR6K*; Cm^R	[28]
pDM4Δ*holD*	pDM4 derivative for the in-frame deletion of *holD* by homologous recombination	This work
pDM4Δ*recA*	pDM4 derivative for the in-frame deletion of *recA* by homologous recombination	[29]

pDM4Δ*polA*	pDM4 derivative for the in-frame deletion of *polA* (PA5493) by homologous recombination	This work
pDM4Δ*polB*	pDM4 derivative for the in-frame deletion of *polB* by homologous recombination	This work
pDM4Δ*dinB*	pDM4 derivative for the in-frame deletion of *dinB* by homologous recombination	This work
pDM4Δ*imuC*	pDM4 derivative for the in-frame deletion of *imuC* by homologous recombination	This work
pDM4Δ*ruvCAB*	pDM4 derivative for the deletion of the *ruvCAB* genes by homologous recombination	This work
pJM253	mini-CTX1 derivative carrying *rhaRS*-P_{rhaBAD}, Tc^R	[27]
pJM253*holD*	pJM253 derivative carrying the coding sequence of *holD* downstream of P_{rhaBAD}	This work
pME6032	IPTG inducible expression vector, $lacI^q$-P_{tac}, Tc^R	[31]
pME*ftsZ-GFP*	pME6032 derivative carrying the coding sequence of *ftsZ* (without the stop codon) fused to the coding sequence of the GFP gene downstream of the IPTG-inducible P_{tac} promoter	This work
pME*polA*	pME6032 derivative carrying the *polA* coding sequence downstream of the IPTG-inducible Ptac promoter	This work
pME*polB*	pME6032 derivative carrying the *polB* coding sequence downstream of the IPTG-inducible Ptac promoter	This work
pME*dinB*	pME6032 derivative carrying the *dinB* coding sequence downstream of the IPTG-inducible Ptac promoter	This work
pME*imuBC*	pME6032 derivative carrying the *imuBC* coding sequences downstream of the IPTG-inducible Ptac promoter	This work
pPS858	pUCP20T derivative, source of the GFP coding sequence for the generation of pME*ftsZ-GFP*	[30]
pFLP2	Broad-host-range plasmid expressing the Flp recombinase, *sacB*; Ap^R/Cb^R	[30]

Table S2. Primers used in this study.[a]

Primer name	Sequence (5'→3')[b]	Restriction site	Application
PA4679_pJM253_FW	ggactagTCGCCTAATTGCCGGTGCC	SpeI	Generation of
PA4679_pJM253_RV	cccaagctTCGCTCATTGAATCTCGCTC	HindIII	pJM253*holD*
PA4679 del_UP_FW	ccgctcgAGCTTCGACGAAGTCACCC	XhoI	
PA4679 del_UP_RV	cgggatCCTGCGGCGCATCGATCG	BamHI	Generation of
PA4679 del_DOWN_FW	cgggatcCGAGCGAGATTCAATGAGCG	BamHI	pDM4Δ*holD*
PA4679 del_DOWN_RV	gctctaGAAGGCCAGCCAGCTACTC	XbaI	
polA del_UP_FW	ccgctcgAGGTCGTAGGGGGCGAGG	XhoI	
polA del_UP_RV	cgggaTCCACCAGGACGAGGGGC	BamHI	Generation of
polA del_DOWN_FW	cgggatCCGCTGGTGGTCGAGGC	BamHI	pDM4Δ*polA*
polA del_DOWN_RV	gctctaGACAAGCTGGCCTTCGGC	XbaI	
polB del_UP_FW	ccgctcgAGCTTCATGTGGCCGTTGC	XhoI	
polB del_UP_RV	cgggatCCCTTGCAGTAACTCCACC	BamHI	Generation of
polB del_DOWN_FW	cgggatCCGGCAGATGGCACTATTC	BamHI	pDM4Δ*polB*
polB del_DOWN_RV	gctctaGACAAGGACGGCGACCTC	XbaI	
dinB del_UP_FW	ccgctcgAGTTCGTCCACTGCCTGGC	XhoI	
dinB del_UP_RV	cgggaTCCGCACCGGAATCTCGC	BamHI	Generation of
dinB del_DOWN_FW	cgggatCCTGCAGGGCGCCCACG	BamHI	pDM4Δ*dinB*
dinB del_DOWN_RV	gctctAGAGTTCATGGAACGCTCGC	XbaI	
imuC del_UP_FW	acgcgtcgaCGCCCCCGTGCATGCCG	SalI	
imuC del_UP_RV	cggaaTTCCGTGGCGCTGGAGGC	EcoRI	Generation of
imuC del_DOWN_FW	cggaatTCACCGGCCTGGACGTCC	EcoRI	pDM4Δ*imuC*
imuC del_DOWN_RV	gctcTAGAGCGTTGGAACCACCTG	XbaI	
ruvC del_UP_FW	ccGCTCGAGGACCTGGACGAC	XhoI	
ruvC del_UP_RV	cgggaTCCGCCACGCTCAACCCC	BamHI	Generation of
ruvB del_DOWN_FW	cgggatCCGGCATTCTCGGCCTGG	BamHI	pDM4Δ*ruvCAB*
ruvB del_DOWN_RV	gCTCTAGAACCAAGTCCCGGC	XbaI	
*ftsZ*_FW	cggaattcAATGTTTGAACTGGTCGATAAC	EcoRI	
*ftsZ*_RV	cccaagcttATCGGCCTGACGACGCAG	HindIII	Generation of
GFP_FW	cccaagcttAGTAAAGGAGAAGAACTTTTCAC	HindIII	pME*ftsZ-GFP*
GFP_RV	ccgctcgagTTATTTGTAGAGCTCATCCATG	XhoI	
*polA*_pME6032_FW	cccgagctCAGGGTTCATTCTACACGC	SacI	Generation of
*polA*_pME6032_RV	cccgagctCGCAACCGTTCATCAGTGG	SacI	pME*polA*
*polB*_pME6032_FW	ggcgagctCGGGTAGAATCGGCGGATG	SacI	Generation of
*polB*_pME6032_RV	ggggtaCCAATGAGCCTCAGAATAGTG	KpnI	pME*polB*
*dinB*_pME6032_FW	GCCCAATGTTAGAATTCAGCG	EcoRI	Generation of
*dinB*_pME6032_RV	cccgagctCTCGCGACTTCGTTGACGC	SacI	pME*dinB*
*imuBC*_pME6032_FW	ccggagctCTGCCAGCCAGCGCCTG	SacI	Generation of
*imuBC*_pME6032_RV	ccggagcTCTTTGTCTCAATGGAAATCCC	SacI	pME*imuBC*
*recN*_RT_FW	AGGAGCAGAAGACCCTGAGC		
*recN*_RT_RV	GACAACAGGTTGACCGCTTC		
*recX*_RT_FW	CCGCTATCTCGAAAGCTTCA		
*recX*_RT_RV	CGCACTCCAGTCGACCTC		
*lexA*_RT_FW	GGCATGAGCATGAAGGACAT		
*lexA*_RT_RV	GAGCGAACTCAGGGTTTTCC		qRT-PCR[c]
*imuB*_RT_FW	GCCATGCTCTTCGAACTGAC		
*imuB*_RT_RV	TGTTCCCAGCCGAGATACTG		
*dinB*_RT_FW	GACTGTTTCTATGCCGCCCT		
*dinB*_RT_RV	CAGATCGGGACACAGCTTGA		
*polA*_RT_FW	TCGATTTCCTCGCCCTGATG		

*polA*_RT_RV	TCGCGATTTTCCTCGAGCTT	
*polB*_RT_FW	CTCGACTTCCTCCTCGAACG	
*polB*_RT_RV	CTTGTAGTCGAGCACCAGCA	
*polC*_RT_FW	GGTTATCGCAACCTCACCGA	
*polC*_RT_RV	GGAACACCTCCATCCACTCG	
*rpoD*_RT_FW	GGGCGAAGAAGGAAATGGTC	
*rpoD*_RT_RV	CAGGTGGCGTAGGTGGAGAA	
M13FW<br>M13RV	GTTTTCCCAGTCACGAC<br>CAGGAAACAGCTATGAC	Sequencing of pBS constructs
P_{rhaBAD}_FW	TCCTGTCAGTAACGAGAAGG	Sequencing of pJM253*holD*
pME6032_FW<br>pME6032_RV	GCTCTCGGGTAACATCAAG<br>CGGTTCTGGCAAATATTCTG	Sequencing of pME6032 constructs
*imuC*_walking 1<br>*imuC*_walking 2	CTTTGGCTGGGCGTCGAG<br>TCATCTCCGAGCAGCCATTG	Sequencing of pME*imuBC*

[a] Preparative PCRs for cloning were performed using the genomic DNA of *P. aeruginosa* PAO1 as the template, with the only exception of the GFP coding sequence that was amplified with primers GFP_FW and GFP_RV using the plasmid pPS858 (Table S1) as the template.

[b] The restriction site used for cloning is underlined in the primer sequence.

[c] When appropriate, these primers have also been used for the sequencing of pME6032 derivatives carrying DNA polymerase genes.

a

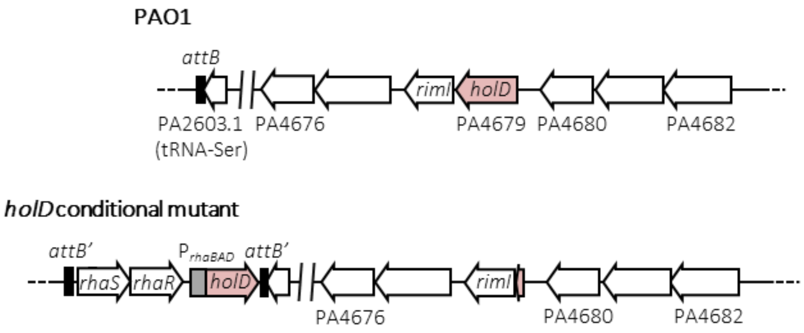

b

```
TGAGGGCGAACCGTCGCATGGCGGGACAAAGCCGCTCCGCCGAGGTCGAAGAACCGCTAT
CATTGCCTGCCTGAACGCCGGGGGCGAAACCGTCCCCGCGCATCGCCTAATTGCCGGTGC
CCGAGATTTGATCGAAGAACAGCGTCGCCGCGCCTTCCTGGGCGCGATGCAGATAACCAG
        M  I  E  E  Q  R  R  R  A  F  L  G  A  M  Q  I  T  S
TTGGCTGCCCCGCCAGCCGTTGCCGTTCGCCGCACCCTCGCGGCCGGAGTTGCTGGAGAC
  W  L  P  R  Q  P  L  P  F  A  A  P  S  R  P  E  L  L  E  T
CCCGCCCCGGGAAGAGCCGGCGGTGCCTGTGCGTGCGCGCGCCGCGATCGATGCGCCGCA
  P  P  R  E  E  P  A  V  P  V  R  A  R  A  A  I  D  A  P  Q
GGCCCGCGTCCCGCAAGCCGAGGCGCCGGCCAGCGCGCCGAGCGTGGCTCCCGCCGCGCC
  A  R  V  P  O  A  E  A  P  A  S  A  P  S  V  A  P  A  A  P
```

Figure S1. (a) Schematic representation of the *P. aeruginosa holD* genetic locus in the wild type strain PAO1 and in the rhamnose-dependent *holD* conditional mutant. (b) DNA sequence (in black) of the upstream and 5' regions of the *holD* gene and amino acid sequence (in red) of the the N-terminal domain of *P. aeruginosa* HolD. The stop codon of the upstream gene (PA4680) is highlighted in red. The start codon demonstrated to encode a functional ψ (HolD) subunit [13] (this study) is highlighted in green, while the start codon annotated in the *Pseudomonas* Genome Database (www.pseudomonas.com), which was shown to provide a non-functional polypeptide *in vitro* [13], is highlighted in yellow.

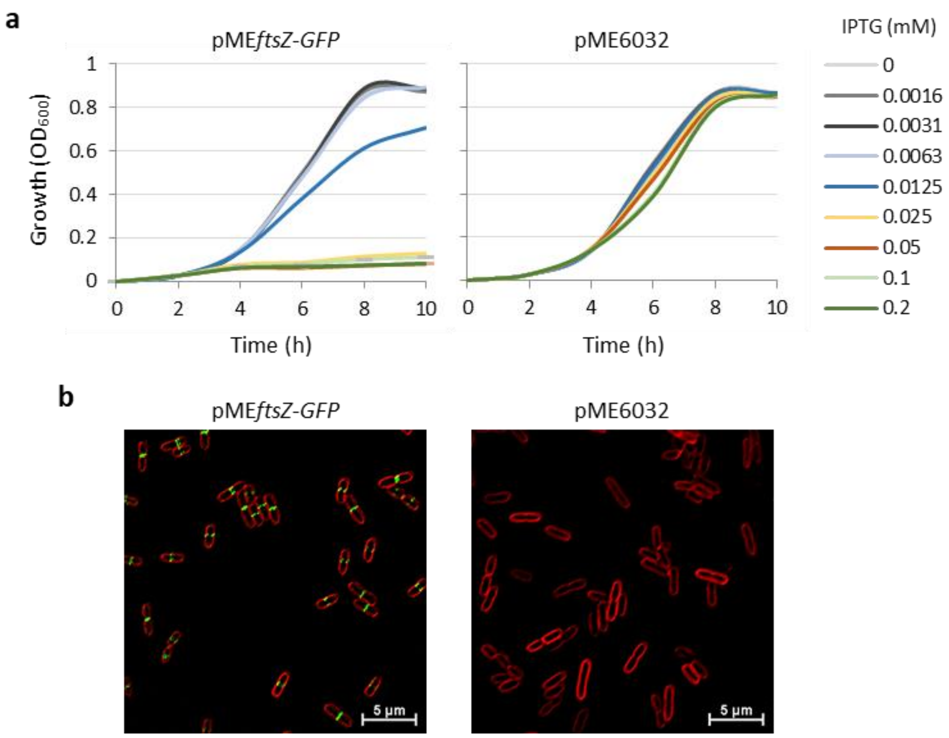

Figure S2. (a) Growth of *P. aeruginosa* PAO1 carrying the construct pME*ftsZ-GFP* or the empty plasmid pME6032 at 37°C in MH in the presence of increasing concentrations of IPTG (0-0.2 mM). Data are the mean of two independent assays. (b) Confocal microscopy images of *P. aeruginosa* PAO1 cells carrying either pME*ftsZ-GFP* or pME6032 cultured as described above in the presence of 0.003 mM IPTG, and stained with the membrane dye FM4-64.

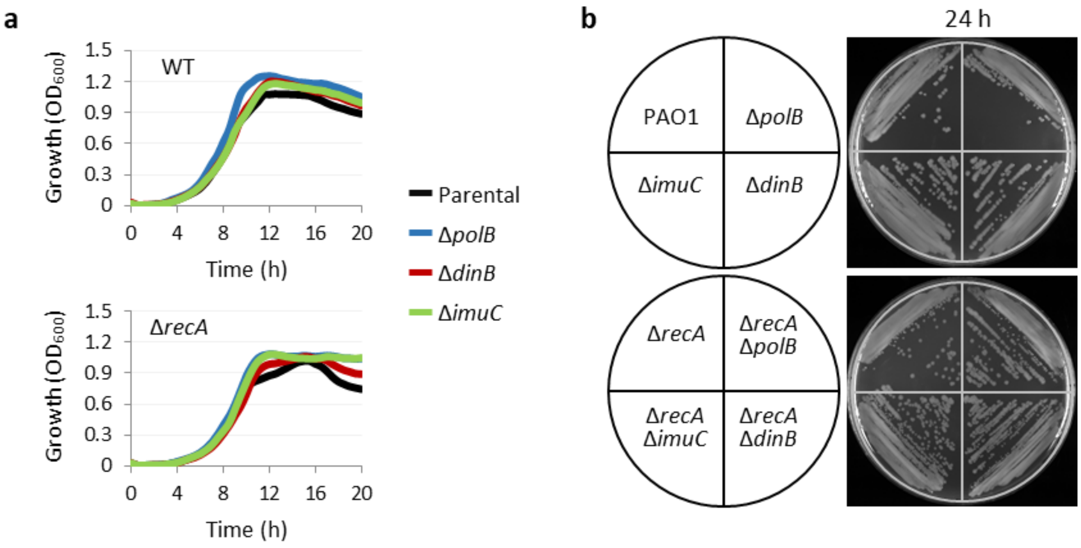

Figure S3. Growth of the *P. aeruginosa* wild type strain PAO1, the Δ*recA* mutant, and their cognate *polB*, *dinB* or *imuC* deletion mutants (a) in MH at 37°C or (b) on MH agar plates after 24 h of incubation at 37°C. Data are the mean and images are representative of three independent assays.

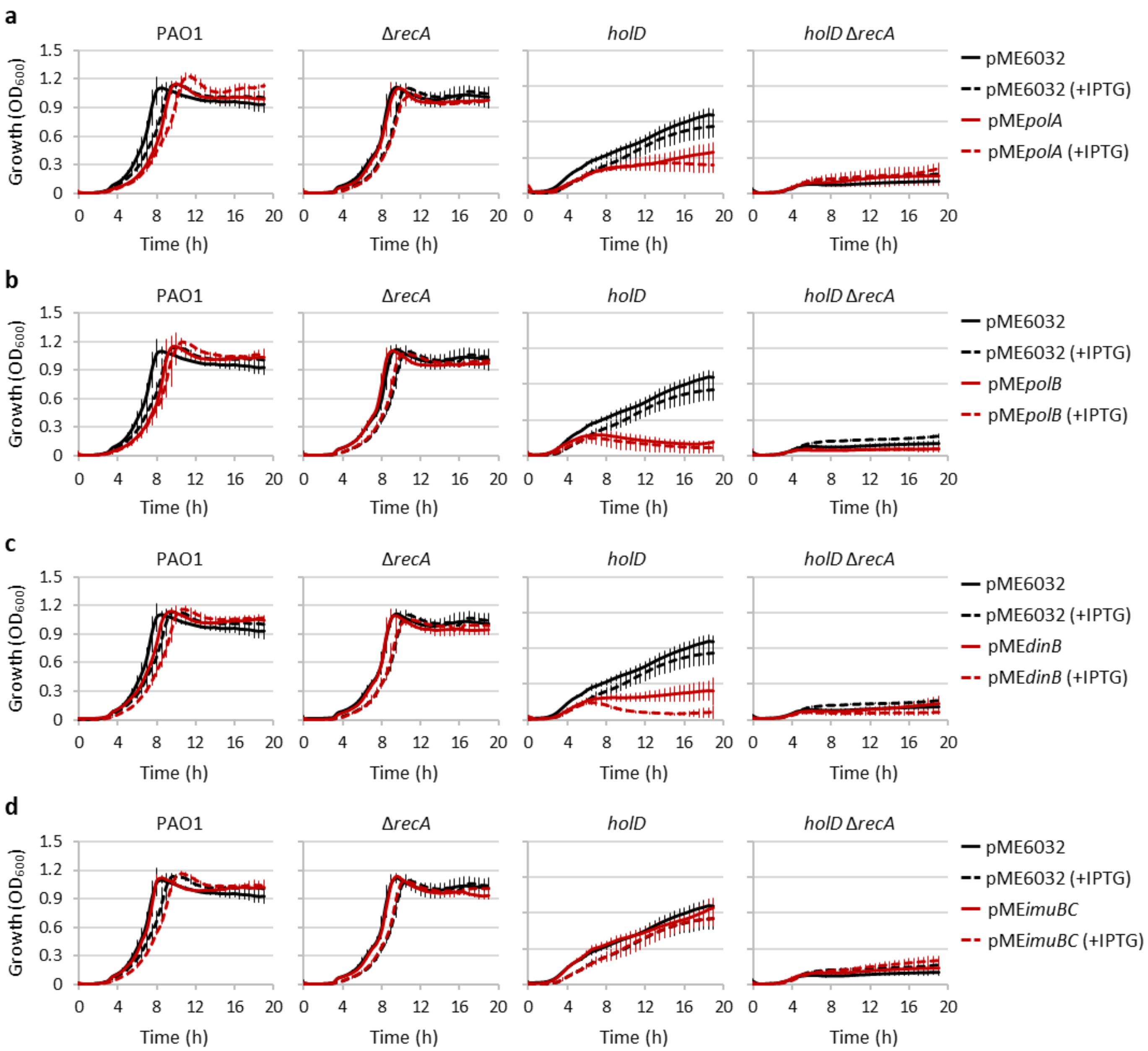

Figure S4. Growth curves of the *P. aeruginosa* wild type (WT) strain PAO1, the *holD* conditional mutant (*holD*), the Δ*recA* mutant, and the *holD* Δ*recA* conditional mutant carrying the empty plasmid pME6032 (black lines) or (a) the PolA-expressing construct pME*polA*, (b) the PolB-expressing construct pME*polB*, (c) the DinB-expressing plasmid pME*dinB*, or (d) the PolA-expressing plasmid pME*polA* (red lines), cultured at 37°C in MH supplemented or not with 0.5 mM IPTG (+IPTG). Data are the mean (±SD) of three independent experiments.
